# A Simple Noninvasive Index Can Predict Hepatocellular Carcinoma in Patients with Chronic Hepatitis B

**DOI:** 10.1038/s41598-017-09358-z

**Published:** 2017-08-21

**Authors:** Lihui Zhu, Tao Li, Xiaomin Ma, Yumin Qiu, Xiaoxiao Ma, Yueke Lin, Lihui Han, Chengyong Qin

**Affiliations:** 1 Shandong University School of Medicine, Jinan, 250012 China; 2 Department of Gastroenterology, Provincial Hospital Affiliated to Shandong University, Jinan, 250021 China; 3 Department of Infectious diseases, Provincial Hospital Affiliated to Shandong University, Jinan, 250021 China; 4 Department of Immunology, Shandong University School of Medicine, Jinan, 250012 China

## Abstract

Screening for possible development of hepatocellular carcinoma (HCC) in patients with chronic hepatitis B (CHB) is essential for risk prediction and early therapy. This study reported a novel model comprised of routine laboratory variables for predicting HCC from CHB. A retrospective study was performed among 463 participants. alpha-fetoprotein (AFP), platelet and alanine aminotransferase (ALT) ratio (APAR) was constructed to differentiate HCC from CHB or non-cancer with area under the receiver operating characteristic curves (AUC) of 0.815 and 0.868 in the training set, 0.831 and 0.861 in the validation set, respectively. In participants with low or normal AFP (<100 ng/mL), the diagnostic efficacy of APAR measured by AUC were 0.817 and 0.809 for predicting HCC from CHB or non-cancer, and at a cutoff of 0.47, the sensitivity, specificity, positive predictive value (PPV), and negative predictive value (NPV) were 89%, 60%, 67% and 86%, respectively. For participants with normal AFP (<20 ng/mL), the AUC of APAR were 0.839 and 0.746 accompanied by a cutoff of 0.36 with sensitivity, specificity, PPV, and NPV of 88%, 69%, 71%, and 87%, respectively. In conclusion, APAR is an effective model for HCC screening especially in those with low even normal serum AFP levels.

## Introduction

Chronic hepatitis B virus (HBV) infection is one of the most threatening factors of developing hepatocellular carcinoma (HCC)^1–3^. Patients with chronic hepatitis B (CHB), even those without cirrhosis are at great risk of developing HCC^4–7^. Screening for potential HCC from CHB patients is essential for risk prediction and decisions on further examinations. A certain number of CHB patients without cirrhosis, especially young adults, are likely to be asymptomatic^8^. Thus diagnosis and treatment for these patients are always been delayed or withheld. On the other hand, HCC patients with early diagnosis, retaining better liver function, can be recommended to surgical radial resection and achieve better prognosis^9, 10^. Thus, it is of great significance to predict early-stage HCC and recognize high-risk patients.

Ultrasonography and alpha-fetoprotein (AFP) test are the most widely used methods for surveillance of HCC in CHB patients^11^. Although ultrasound surveillance is in general effective and harmless, it is limited by central obesity, experience and skill of operator as well as the fact that it cannot distinguish small hepatocellular carcinoma (SHCC) and liver cirrhotic nodules^12, 13^. Moreover, ultrasound is not available in some remote areas. In addition, some HCC patients keep persistent low even normal AFP levels during whole course, while some patients with chronic liver disease, such as CHB and chronic hepatitis C (CHC), turn up elevated AFP levels^6, 14, 15^. Hence, it’s necessary to develop more accurate and reliable noninvasive means to supplement present ultrasonography surveillance and watch out for potential HCC more efficiently.

HCC is a systematic disease and needs to be judged from an integrated point of view. HCC cells actively proliferate and secrete plentiful AFP, thus elevating the serum AFP level^16^. HCC cells multiply and destroy normal parenchymal cells causing disorder of lobe structure and changes of main enzymology indexes which reflect liver dysfunction^17^. Abnormal changes of liver result in portal system diseases, such as portal vein varicose, splenauxe, and hypersplenism, leading to variation of blood cells composition. On account of the systemic reactions caused by HCC, comprehensive index can be superior to single index, which can predict the occurrence of HCC with high accuracy. Routine laboratory variables are much more meaningful and readily accessible, since liver biochemical examination, blood routine examination and AFP test are routine inspections for each CHB outpatient. An ideal approach for predicting HCC would be non-invasive, accurate, accessible and inexpensive. An index comprising routinely laboratory variables would meet these criteria. The aim of our research is to develop such a simple model comprised of routine laboratory variables for predicting HCC from CHB patients, especially those with low even normal serum AFP levels.

To accomplish this, clinical and laboratory data collected from a training set of 224 consecutive patients were used to formulate the predictive model, which were validated in an independent set of 239 subsequent patients. Our results showed that AFP, platelet (PLT) and alanine aminotransferase (ALT) ratio (APAR) performs well as a model to distinguish HCC patients from those with nonmalignant CHB.

## Results

### Patient characteristics

**Figure 1:**
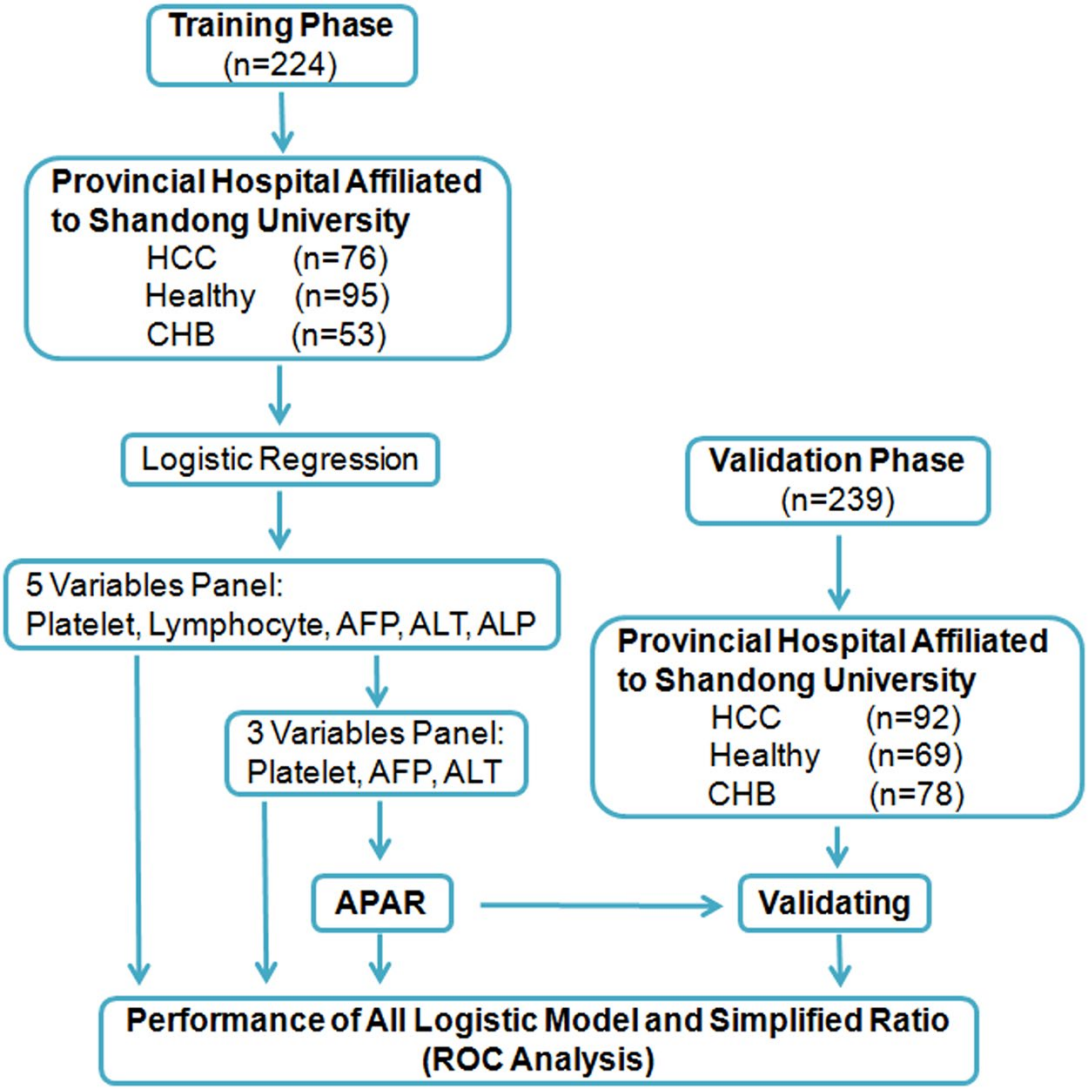
Study design. ALP, alkaline phosphatase; ROC, receiver operating characteristics; APAR: 1000 × AFP [ng/ml]/(PLT[×10^9^/L] × ALT[U/L]).

There were two phases included in our study (Fig. 1). The characteristics of participants randomized to the training and validation sets are shown in Table 1. This study included three cohorts for investigation, HBV-related HCC patients, patients with CHB and the healthy. The mean values for the whole cohort were: sex (79% male), age (50 ± 13 years), white blood cell (WBC) count (5.6 ± 2.0 × 10^9^/L), red blood cell (RBC) count (4.6 ± 0.7 × 10^12^/L), PLT count (182 ± 84 × 10^9^/L), lymphocyte count (1.7 ± 0.7 × 10^9^/L), monocyte count (0.4 ± 0.2 × 10^9^/L), neutrophil count (3.2 ± 1.6 × 10^9^/L), AFP level (180.2 ± 404.8 ng/ml), aspartate aminotransferase (AST) (118 ± 204 U/L), ALT (169 ± 366 U/L), gamma-glutamyl transpeptidase (GGT) (98 ± 135 U/L), alkaline phosphatase (ALP) (119 ± 66 U/L), total bilirubin (TBIL) (46.6 ± 81.0 umol/L). In the recruited cohorts of participants, there were no significant differences in the distribution of RBC, PLT, lymphocyte, monocyte, GGT, ALP, ALT and AFP between the training and validation sets (p > 0.05). Total BCLC stages of HCC were also well balanced between the training and validation sets.

**Table 1:** Comparison of characteristics of patients with HCC, CHB and healthy control in training and validation sets.

Variables	Training Set (n = 224)			Validation Set (n = 239)		
	HCC (n = 76)	Healthy (n = 95)	CHB (n = 53)	HCC (n = 92)	Healthy (n = 69)	CHB (n = 78)
Gender						
Male (%)	58 (76)	58 (61)	40 (75)	75 (82)	54 (78)	61 (78)
Female (%)	18 (24)	37 (39)	13 (25)	17 (18)	15 (22)	17 (22)
Age, years	57 ± 9	45 ± 10	46 ± 14	58 ± 8	53 ± 13	39 ± 12
White cell count (×10^9^/L)	5.0 ± 2.0	6.2 ± 1.5	5.7 ± 2.4	4.4 ± 2.1	6.3 ± 1.1	5.9 ± 2.1
Red blood count (×10^12^/L)	4.3 ± 0.8	5.0 ± 0.5	4.5 ± 0.7	4.3 ± 0.8	5.0 ± 0.5	4.7 ± 0.7
Platelet count (×10^9^/L)	132 ± 73	251 ± 51	165 ± 82	121 ± 67	250 ± 50	169 ± 64
Lymphocyte (×10^9^/L)	1.3 ± 0.7	2.3 ± 0.7	1.7 ± 0.6	1.2 ± 0.6	2.1 ± 0.4	1.8 ± 0.6
Monocyte (×10^9^/L)	0.4 ± 0.2	0.4 ± 0.1	0.5 ± 0.3	0.4 ± 0.2	0.4 ± 0.1	0.5 ± 0.2
Neutrophil (×10^9^/L)	3.1 ± 1.5	3.3 ± 1.1	3.3 ± 2.0	2.7 ± 1.8	3.5 ± 1.0	3.4 ± 2.0
AFP (ng/ml)	372.6 ± 475.9	3.2 ± 1.6	94.4 ± 177.0	423.1 ± 645.9	3.4 ± 2.0	136.7 ± 260.6
AST (U/L)	82 ± 114	21 ± 7	316 ± 319	68 ± 110	24 ± 10	277 ± 277
ALT (U/L)	67 ± 101	22 ± 13	525 ± 716	49 ± 41	24 ± 18	474 ± 439
GGT (U/L)	147 ± 202	33 ± 23	129 ± 97	111 ± 134	30 ± 17	155 ± 157
ALP (U/L)	145 ± 74	83 ± 21	121 ± 61	148 ± 94	85 ± 21	131 ± 52
TBIL (umol/L)	37.1 ± 62.0	13.7 ± 5.8	104.8 ± 126.6	34.2 ± 40.1	15.6 ± 6.2	98.2 ± 124.0
BCLC stages						
0 (%)	5 (7)			11 (12)		
A (%)	18 (24)			25 (27)		
B (%)	23 (30)			26 (28)		
C (%)	30 (39)			29 (32)		
D (%)	0 (0)			1 (1)		

Values are expressed as mean ± SD. GGT, gamma-glutamyl transpeptidase; TBIL, total bilirubin.

### Candidate predictors for HCC from training set

**Figure 2:**
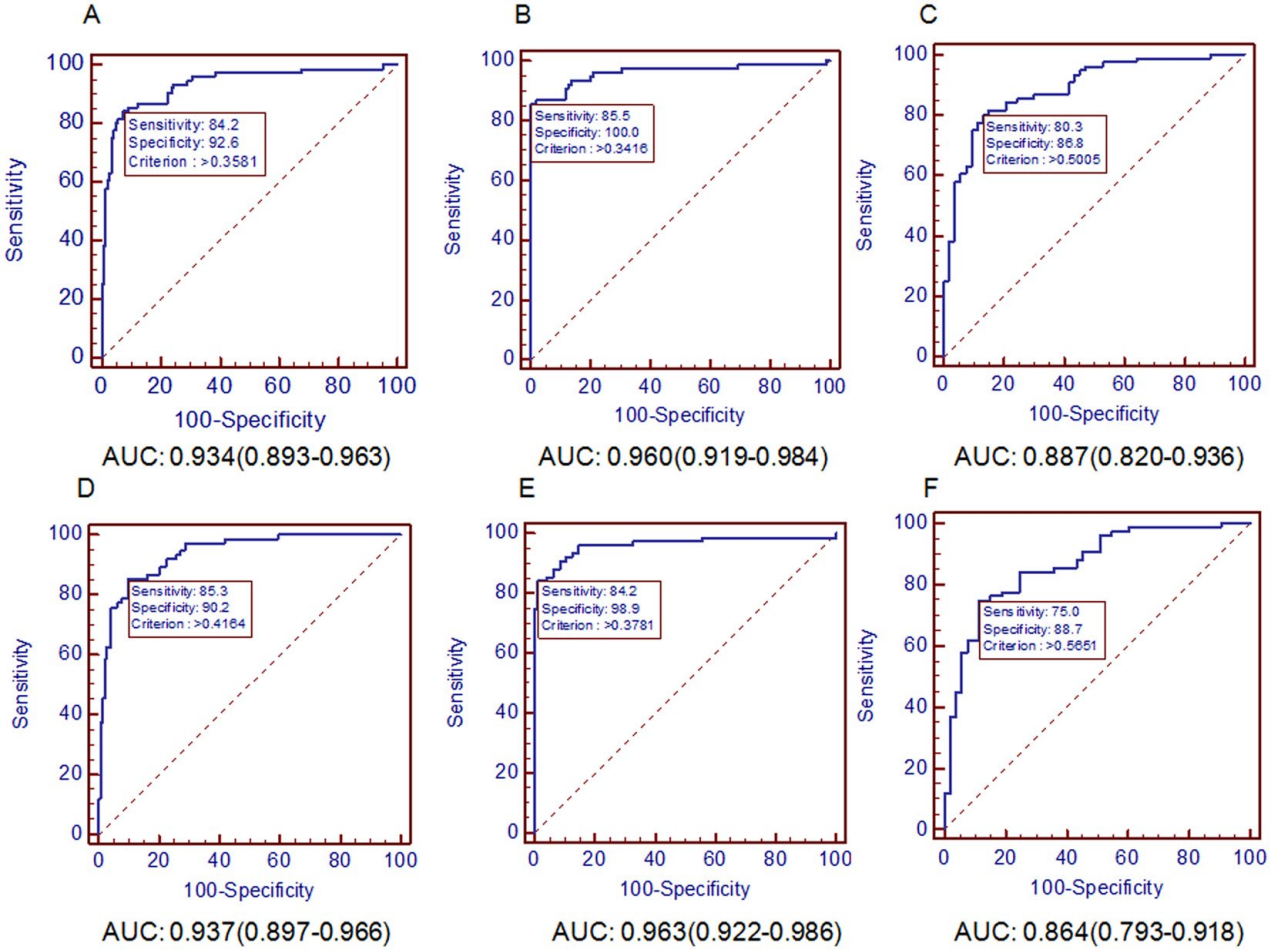
ROC analysis for HCC diagnosis in the training set. AUC of 5-variable panel for predicting HCC in (**A**) HCC versus non-cancer control in the training set, (**B**) HCC versus healthy in the training set, (**C**) HCC versus CHB in the training set. AUC of 3-variable panel for predicting HCC in (**D**) HCC versus non-cancer control in the training set, (**E**) HCC versus healthy in the training set, (**F**) HCC versus CHB in the training set.

In the training set (n = 224), CHB patients and healthy participants were classified as non-cancer control. When comparing HCC patients with non-cancer control, univariate analysis revealed significant different expressions of WBC count, RBC count, PLT count, lymphocyte count, AFP, ALT, GGT and ALP. No significant differences were observed in monocyte count, neutrophil count, AST and TBIL (Table 2). Multivariate logistic regression analysis identified 5 variables as independent predictors of HCC: PLT count, lymphocyte count, AFP, ALT and ALP (Table 2). Receiver operating characteristic (ROC) curves of 5-variable panel for predicting HCC from non-cancer control, the healthy, and CHB patients in the training set were plotted in Fig. 2A–C with area under the curves (AUC) of 0.934, 960 and 0.887, respectively. To further make the panel more simple and applicable, we simplified the 5-variable panel into a 3-variable panel including PLT count, AFP and ALT (Table 2). As shown in Fig. 2D–F, the AUC of 3-variable panel for predicting HCC from non-cancer control, the healthy, and CHB patients in the training set were 0.937, 0.963 and 0.864. Hence, the 3-variable panel can also predict HCC with similar diagnostic efficacy as the 5-variable panel with simplification.

**Table 2:** Univariate and multivariate analysis of routine laboratory data in the training set associated with further analysis of risk factors.

Routine laboratory data	HCC versus Non-cancer Control^a^							
	Univariate		Multivariate			Further^b^		
	P	Mean ± SD	B	S.E.	P	B	S.E.	P
White cell count (×10^9^/L)	<0.001	(5.0 ± 2.0 versus 6.0 ± 1.9)						
Red blood count (×10^12^/L)	<0.001	(4.3 ± 0.8 versus 4.8 ± 0.6)						
Platelet count (×10^9^/L)	<0.001	(132 ± 73 versus 220 ± 76)	−0.012	0.003	0.001	−0.02	0.003	<0.001
Lymphocyte (×109/L)	<0.001	(1.3 ± 0.7 versus 2.1 ± 0.7)	−1.07	0.398	0.007			
Monocyte (×10^9^/L)	0.524	(0.4 ± 0.2 versus 0.5 ± 0.2)						
Neutrophil (×10^9^/L)	0.362	(3.1 ± 1.5 versus 3.3 ± 1.5)						
AFP (ng/ml)	<0.001	(372.6 ± 475.9 versus 35.9 ± 114.1)	0.004	0.001	<0.001	0.01	0.001	<0.001
AST (U/L)	0.134	(82 ± 114 versus 127 ± 237)						
ALT (U/L)	0.04	(67 ± 101 versus 202 ± 490)	−0.006	0.002	0.002	−0.01	0.002	0.003
GGT (U/L)	<0.001	(147.3 ± 202.3 versus 67.2 ± 76.6)						
ALP (U/L)	<0.001	(144.8 ± 74.0 versus 96.4 ± 44.1)	0.017	0.005	0.002			
TBIL (umol/L)	0.417	(37.1 ± 62.0 versus 46.3 ± 87.2)						

^a^Non-cancer Control group includes healthy participants and patients with CHB. ^b^Further analysis means for simplification, the 5-variable panel was simplified to a 3-variable panel without losing accuracy after repeated verification.

### APAR was proposed as a novel model for HCC diagnosis

**Figure 3:**
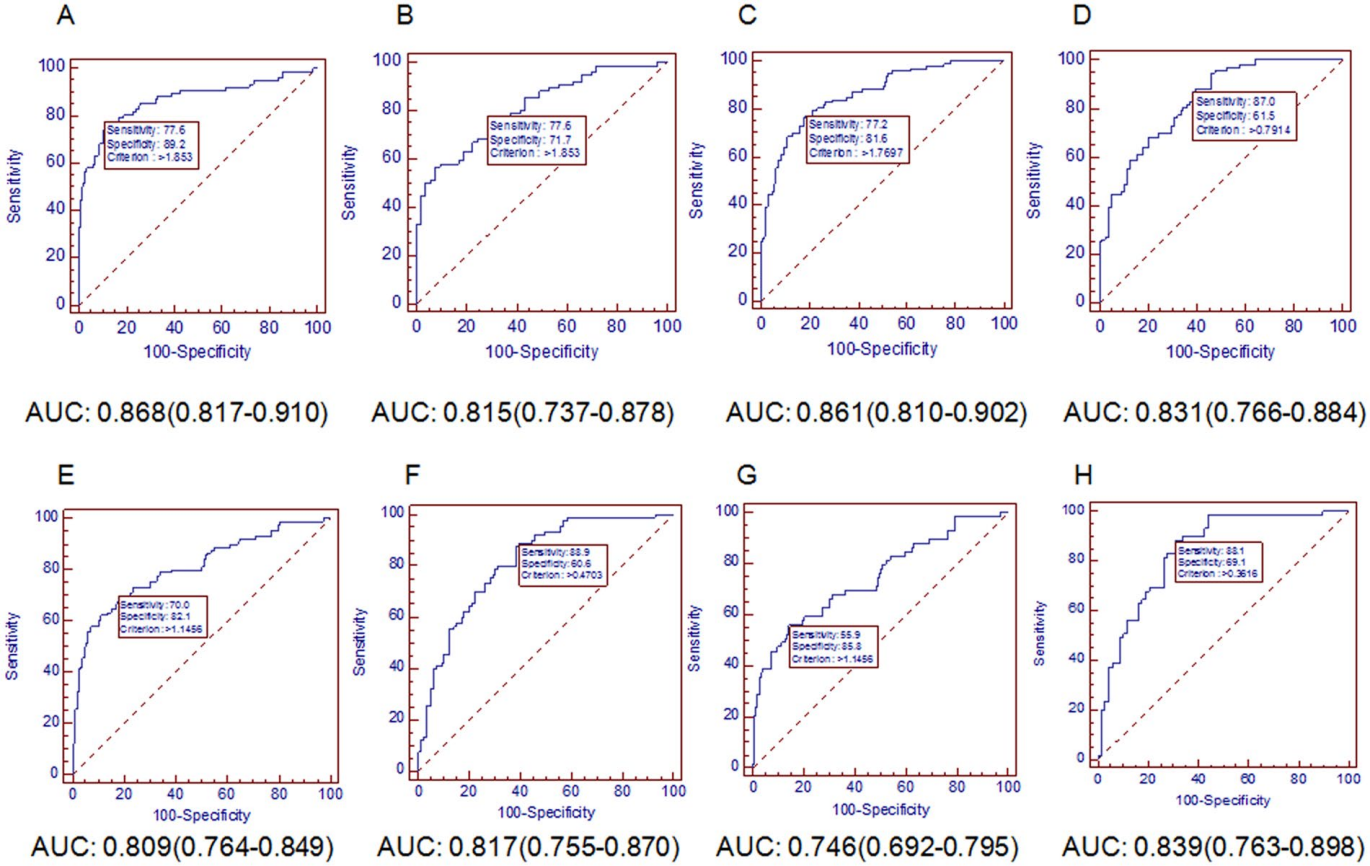
ROC analysis of APAR for HCC diagnosis. AUC of APAR for predicting HCC in (**A**) HCC versus non-cancer control in the training set, (**B**) HCC versus CHB in the training set, (**C**) HCC versus non-cancer control in the validation set, (**D**) HCC versus CHB in the validation set, (**E**) HCC versus non-cancer control in the whole set of participants of low or normal AFP (<100 ng/mL), (**F**) HCC versus CHB in the whole set of participants of low or normal AFP (<100 ng/mL), (**G**) HCC versus non-cancer control in the whole set of participants of normal AFP (<20 ng/mL), (**H**) HCC versus CHB in the whole set of participants of normal AFP (<20 ng/mL).

In the 3-variable panel, AFP level was significantly elevated whereas the PLT count and the ALT level were decreased from non-cancer control to HCC. Based on relationship of the 3 parameters, the following simple index called AFP, PLT and ALT ratio (APAR): 1000 × AFP[ng/ml]/(PLT[×10^9^/L] × ALT[U/L]) were derived. The results of APAR in differentiating HCC from non-cancer control or CHB patients in the training and validation sets are shown in Fig. 3A–D. In the training set the AUC for APAR in differentiating HCC from non-cancer control (0.868) was higher compared with its ability to differentiate HCC from patients with CHB (0.815) (Fig. 3A,B). Applying APAR in the validation set gave a slightly lower AUC in differentiating HCC from non-cancer control (0.861) and a slightly higher AUC in predicting HCC from patients with CHB (0.831) than the training set (Fig. 3C,D).

Based on the ROC curves of APAR, an optimized cut-off value of 1.85 was certified to distinguish HCC from CHB (Table 3). Using cut-off value of ≥ 1.85 in both training set and validation set, 76.2% of the CHB patients with an APAR ≥ 1.85 had developed HCC (i.e., positive predictive value (PPV) = 76.2%), whereas an APAR < 1.85 had a sensitivity of 76.2% and a negative predictive value (NPV) of 69.5%. At the cut-off value of 1.85 in the training set, 77 (34.4%) participants had elevated APAR (≥1.85) while 147 participants had lower APAR (<1.85). Among the patients with APAR of 1.85 or greater, 59 of 77 (76.6%) participants were patients with HCC. Simultaneously, for patients with APAR less than 1.85, 130 of 147 (88.4%) participants were non-cancer control, only 17 (11.6%) HCC patients were falsely classified. Meanwhile, the cut-off value for APAR of 1.85 was verified in the validation set. Similarly, in the higher APAR group (≥1.85), 69 of 95 (72.6%) participants were patients with HCC. On the other hand, in the lower APAR group (<1.85), 122 of 144 (84.7%) participants were non-cancer control, only 22 (15.3%) HCC patients were classified incorrectly. Together, using APAR cut-off value (1.85) to screen HCC development in CHB patients was high-efficiency, even a small number of CHB were incorrectly divided into HCC and can be ruled out through further examinations.

**Table 3:** Accuracy of the APAR index in predicting HBV-related HCC in different cohorts of participants.

Group(N)	APAR	CHB	HBV-related HCC	Sensitivity	Specificity	PPV	NPV
Total^a^ (299)	<1.85	91	40	76.20%	69.50%	76.20%	69.50%
	≥1.85	40	128				
Low or normal AFP^b^ (189)	<0.47	59	10	88.90%	59.60%	66.70%	85.50%
	≥0.47	40	80				
Normal AFP^c^ (127)	<0.36	47	7	88.10%	69.10%	71.20%	87.00%
	≥0.36	21	52				

^a^Total cohort includes patients with CHB and HBV-related HCC in the training and the validation set. ^b^Participants with low or normal AFP includes CHB patients and HBV-related HCC in the whole set of participants with AFP < 100 ng/mL. ^c^Participants with low AFP includes CHB patients and HBV-related HCC in the whole set of participants with AFP < 20 ng/mL.

### APAR has a good diagnostic efficacy for HBV-related HCC patients with low even normal AFP levels

The accuracy of APAR for predicting HCC from CHB was further detected in the whole set of participants with low or normal AFP level (<100 ng/mL). The diagnostic efficacy of APAR measured by AUC for predicting HCC from CHB patients (0.817, Fig. 3F) is higher than its ability to predict HCC from non-cancer control (0.809, Fig. 3E).

At a cutoff of <0.47, the NPV to exclude HCC from CHB was 86% with a sensitivity of 89%, meanwhile ≥0.47 had a PPV of 67% with a specificity of 60% (Table 3). In the whole set of participants with low or normal AFP (<100 ng/mL), there were 120 (63.5%) patients in the higher APAR group (≥0.47) and 69 (36.5%) in the lower APAR group (<0.47). What’s more, 80 of 120 (66.7%) patients with higher APAR (≥0.47) were HCC patients, and 59 of 69 (85.5%) patients in the lower APAR group (<0.47) were no-cancer control.

Then we explored the diagnostic efficiency of APAR in the whole set of participants with normal AFP level (<20 ng/mL). Surprisingly, the diagnostic efficiency of APAR for distinguishing HCC from CHB in patients with normal AFP (0.839, Fig. 3H) come top among its diagnostic ability to distinguish HCC from CHB in sets with entire range of AFP (0.815 in the training set, Fig. 3B; 0.831 in the validation set, Fig. 3D) or in set with low or normal AFP (<100 ng/mL) (0.817, Fig. 3F). This result might implicate that APAR has higher application value in patients with normal AFP. However, its ability to differentiate HCC from non-cancer control decreased (0.746, Fig. 3G). Based on the AUC, a cutoff of <0.36 had a NPV of 87% with a sensitivity of 88%, meanwhile ≥0.36 had a PPV of 71% with a specificity of 69% (Table 3). Paying close attention to the higher APAR group (≥0.36), 52 of 73 (71.2%) were patients with HCC. In patients with lower APAR (<0.36), 47 of 54 (87.0%) were CHB.

### APAR has a good diagnostic efficacy for HCC patients with different BCLC stages

**Figure 4:**
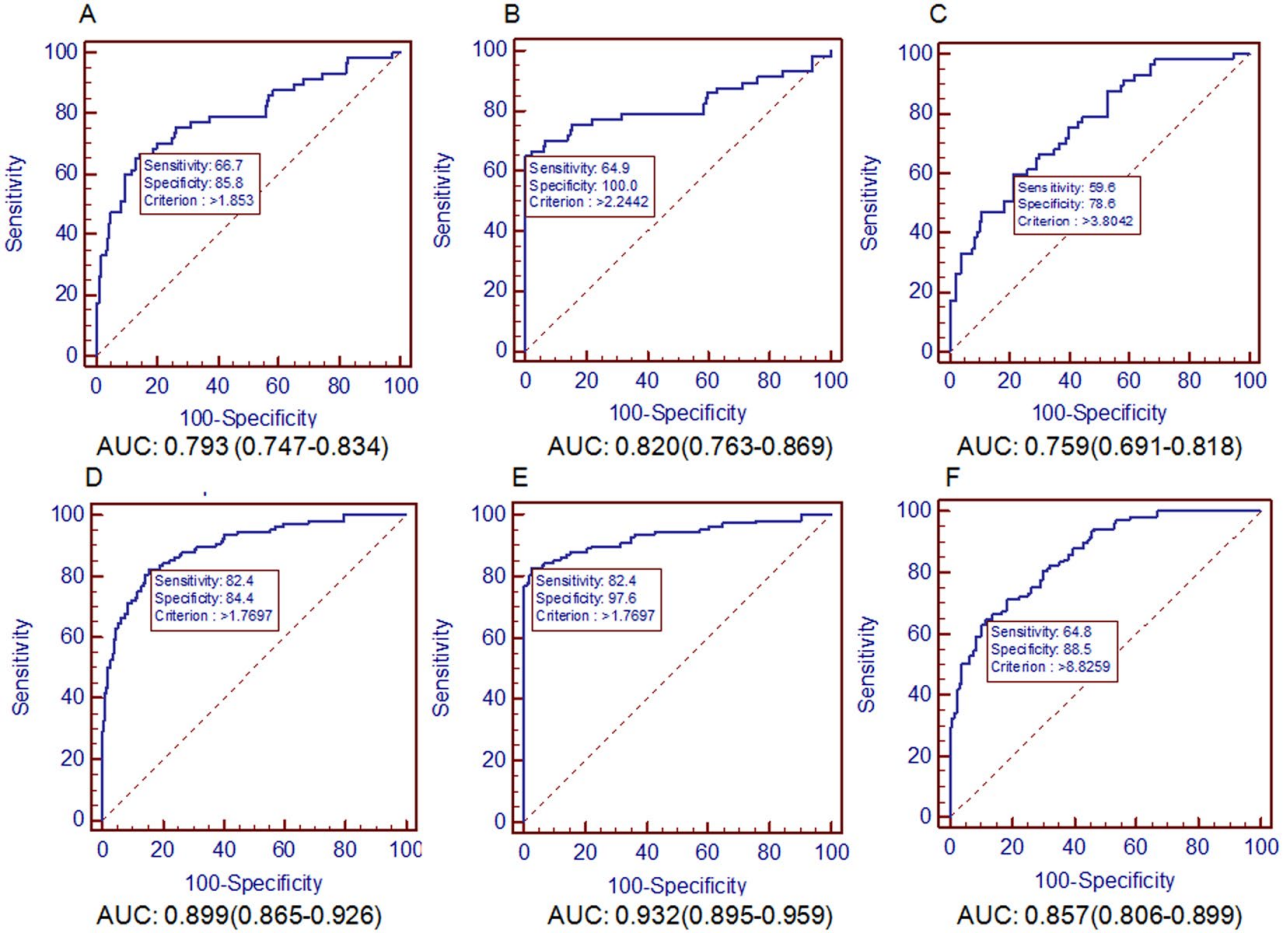
ROC analysis of APAR for HCC diagnosis in different BCLC stages. AUC of APAR for predicting HCC in (**A**) HCC versus non-cancer control in the early BCLC stages, (**B**) HCC versus healthy in the early BCLC stages, (**C**) HCC versus CHB in the early BCLC stages; (**D**) HCC versus non-cancer control in the late BCLC stages, (**E**) HCC versus healthy in the late BCLC stages, (**F**) HCC versus CHB in the late BCLC stages.

The diagnostic efficiency of APAR in differentiating HCC patients with different BCLC stages including early stages (0 and A) and advanced stages (B, C, and D) from the non-cancer control, healthy, and CHB was also evaluated, respectively (Fig. 4). The analysis displayed that the APAR also can discriminate HCC with early stages from non-cancer control (AUC = 0.793; 95% CI, 0.747 to 0.834; sensitivity = 66.7%, specificity = 85.8%), the healthy (AUC = 0.820; 95% CI, 0.763 to 0.869; sensitivity = 64.9%, specificity = 100.0%), and CHB patients (AUC = 0.759; 95% CI, 0.691 to 0.818; sensitivity = 59.6%, specificity = 78.6%). In addition, APAR also can differentiate HCC with advanced stages from these controls. The corresponding AUC were 0.899 (95% CI, 0.865 to 0.926; sensitivity = 82.4%, specificity = 84.4%), 0.932 (95% CI, 0.895 to 0.959; sensitivity = 82.4%, specificity = 97.6%) and 0.857 (95% CI, 0.806 to 0.899; sensitivity = 64.8%, specificity = 88.5%), respectively. These results suggest that APAR has a clinical base and can also predict some clinical characteristics of HCC patients in a certain degree, especially advanced pathological features.

## Discussion

Neoplastic diseases could give rise to changes of various indexes, as a result that a comprehensive model comprised of various traditional indexes is expected to improve the diagnostic efficacy of potential neoplastic diseases^18, 19^. Currently, in diagnosis of HCC, the diagnostic markers used in clinical practice are mostly organ specific single indicators, for example, the most common available serum marker AFP and protein induced by vitamin K absence or antagonist-II (PIVKA-II) in the surveillance and diagnosis of HCC^20–22^. However, these indicators, along with false positive or false negative sometimes^6, 23, 24^, cannot meet the need of clinical diagnosis, especially early diagnosis. Several studies have introduced ratios using the routine laboratory variables for prediction of HCC. Neutrophil to lymphocyte ratio (NLR) was used to predict survival of HCC, prognosis of HCC patients after liver resection, recurrence after liver transplantation, recurrence after radiofrequency ablation^25–29^. PLT to lymphocyte ratio (PLR) acts as a prognostic factor for advanced HCC patients and patients with huge HCC that received transarterial chemoembolization (TACE)^30^. AST to PLT ratio predicts prognosis for hepatitis B-induced HCC after hepatic resection^31^. Many studies combined several indicators to predict survival of HCC^32, 33^. However, none of these researches explored surveillance capacity of these ratios for HBV-related HCC, especially from the high risk population with chronic HBV infection. In consequence, novel model for HCC surveillance and diagnosis in high risk population are urgently needed. Hence, our study devoted to establish an index for predicting the occurrence of HBV-related HCC. In this study, we derived a novel model, AFP to PLT and ALT ratio (APAR) for diagnosis and surveillance of HCC in CHB patients. Especially, APAR has good diagnostic efficiency in HCC patients with low or even normal AFP levels.

Our study has several unique features. Firstly, we had a relatively large cohort comprising 463 participants which guaranteed a comparable higher accuracy. And that we had two independent groups of participants dividing into the training set and the validation set to ensure the authenticity and conviction. Secondly, our study included the healthy and CHB patients together to comprise non-cancer control in order to avoid missing certain indicators, so as to improve the sensitivity and specificity for predicting HCC from CHB or the healthy, respectively. In addition, we did all of the diagnostic analysis of HCC from non-cancer control, the healthy, as well as the high risk population with chronic HBV infection. Finally, we conducted the analysis of APAR in the patients with low and normal AFP level, and we found that APAR has a good diagnostic efficacy for HBV-related HCC patients with low even normal AFP levels.

Ultrasonography is still an effective and commonly used method for HCC surveillance by providing useful imaging of a potential HCC lesions^11^. However, the efficiency of diagnosis is limited by the many factors, such as operator experience, the device quality, the size of lesions and so on. For example, ultrasonography is inability to distinguish small HCC lesions from cirrhotic lesions (especially in size range ≤20 mm)^12, 13^, and result in low sensitivity in diagnosing early-stage HCC^12, 13, 34^. Improvement of HCC diagnosis efficiency of ultrasonography is one of the problems needed to be addressed. AFP levels is a most common used method for HCC surveillance, however, there are some HCC patients whose AFP levels constantly stay low or normal in clinical practice, and some patients with CHB and cirrhosis whose AFP elevated as false positive. According to our study, the diagnostic efficiency of APAR (AUC = 0.817) was better than AFP (AUC = 0.532) in CHB patients with low or normal AFP with a sensitivity of 88.9%. The diagnostic efficiency of APAR (AUC = 0.839) can be significantly elevated when it applied in patients with normal AFP. Therefore, APAR improves the diagnostic efficiency of the single marker AFP, and can serve as alternative to AFP for screening HCC before ultrasonography, especially in patients with low and normal AFP levels. CHB patients whose ultrasonic imaging are not precisely defined can be preliminarily identified by APAR and can be admitted for further examinations (including CT and MRI) for identification or more frequent follow-up monitoring. The components of APAR are obtained from routine hematologic examinations for CHB patients. Thus, no additional tests are needed for this analysis, making it convenient for clinical application.

AFP is widely used as a HCC serum marker^11^. The combination of PLT and ALT, is predominantly reflection of hepatic cirrhosis (similar to AST-to-PLT ratio index (APRI) with respect to hepatic cirrhosis)^35^, so the APAR value represent a reflection of systemic conditions. HCC is a systematic disease and needs to be judged from an integrated point of view. These features of APAR may account for its superiority in identifying high-risk patients to recognize early-stage HCC more effectively based on the fact that this metric reflects a system response to the development of HCC. High APAR values can be recognized as a “warning-signal” in the patients especially in whose ultrasonic imaging of lesion is indistinguishable. From this perspective, APAR can assist clinicians by preliminarily identifying the set of patients at particular risk of development of HCC and can be used as a powerful supplement to ultrasonography.

The diagnostic efficacy of APAR for predicting HCC has superiority than AFP in diagnosing HCC from non-cancer control, as well as from CHB patients (Table 4). To further investigate clinical significance of APAR, we further analyzed the diagnostic efficacy of APAR in different BCLC stages of HCC patients. Our data showed that the diagnostic efficacy of APAR is stable and efficient in all stages of HCC patients, which indicated that APAR is a constant marker for all types of HBV-related HCC.

**Table 4:** Comparison of AUC between APAR and AFP for predicting HCC from non-cancer control or CHB patients.

	Training Set		Validation Set		Whole set with normal AFP (<20 ng/uL)		Whole set with low or normal AFP (<100 ng/uL)	
	HCC vs. Control	HCC vs. CHB	HCC vs. Control	HCC vs. CHB	HCC vs. Control	HCC vs. CHB	HCC vs. Control	HCC vs. CHB
	AUC(95%CI)	AUC(95%CI)	AUC(95%CI)	AUC(95%CI)	AUC(95%CI)	AUC(95%CI)	AUC(95%CI)	AUC(95%CI)
APAR	0.868(0.817–0.910)	0.815(0.737–0.878)	0.861(0.810–0.902)	0.831(0.766–0.884)	0.746(0.692–0.795)	0.839(0.763–0.898)	0.809(0.764–0.849)	0.817(0.755–0.870)
AFP	0.815(0.752–0.878)	0.646(0.553–0.740)	0.739(0.673–0.805)	0.604(0.520–0.689)	0.633(0.547–0.719)	0.435(0.334–0.536)	0.683(0.632–0.732)	0.532(0.459–0.605)

The association between increased APAR value and malignant outcome is complex and remains to be elucidated. The APAR is comprised of AFP level, PLT count and ALT level. There are several possible reasons accounting for this positive correlation which may be explained from the involved individual parameters from the following aspects. (1) AFP was a predictor of HCC that had been proved in many studies and its inaccuracy was also mentioned in some studies^14, 36^. (2) As for the PLT count, we supposed that on the one hand the production of PLT was reduced in HCC patients for the reason that the destruction of hepatocytes leading to the reduction of the thrombopoietin^37^. On the other hand, the destruction of PLT will be increased on account of the enlarging spleen. Due to anatomy structure changing of liver and the present of tumor thrombus in portal vein, HCC patients always has the symptom of portal hypertension, which leads to congestion, enlarging and structural changing of the spleen and often associated with hypersplenism. (3) HCC patients often have the complication of thrombocytopenia syndrome which leads to the decrease of PLT count. ALT will increase during the CHB since that hepatocytes release ALT to serum after the HBV damaging hepatocytes. However, ALT will decrease during HCC without the hepatitis reaction.

Although, a considerable proportion of HCC patients (11.6% in the training set and 15.3% in the validation set) are really misclassified by the APAR score, this simple index is also a potential useful method by identifying the high risk patients with HCC in clinical practice, with positive predictive value of 76.2% and negative predictive value of 69.5% at 1.85 cut-off point. Actually, in this kind of simple noninvasive indexes, APAR has a fairly high diagnostic efficiency, which is measured by AUCs of 0.868 and 0.815 in the training set and 0.861 and 0.831 in the validation set in distinguishing HCC patients from non-cancer controls and CHB patients, respectively. By contrast, the diagnostic efficiencies of APRI-B, PLT-B and AST-B, in predicting HCC (the AUCs were 0.715, 0.733 and 0.609, respectively)^38^ are much lower than that of APAR. Another index, which consists of AFP, cytokeratin-19 and glypican-3 for prediction of early recurrence of HCC, only has an AUC of 0.767^39^. Therefore, the diagnostic efficiency of APAR can be acceptable in clinical practice. Patients who were misdiagnosed as HCC by APAR, can receive further examinations, such as CT or MRI, in order to be excluded from HCC eventually. This price is not too high to paid, when compared with the serious consequence of missed diagnosis of HCC. Additionally, enhancing the sensitivity of APAR is also needed for the decrease of missed diagnosis of HCC.

Based on the current data, in the set of patients with cirrhosis, having the highest risk of HCC, the performance of APAR in differentiating HCC patients from cirrhosis patients is not better than AFP (see Supplementary Fig. S1). Hepatic cirrhosis is regarded as “precancerous lesions” of HCC. Giving for the sake of this severe situation, all cirrhotic patients are highly recommended for HCC surveillance by CT and/or MRI inspections according to the current guidelines^40^. Comparatively, CHB patients with undefined cirrhosis, whose chance of canceration is relatively low, were more likely to be ignored for precise examinations for early diagnosis. APAR index is mainly suggested to be used in these CHB patients. As a “warning-signal”, high APAR value can remind the clinicians to avoid this missed diagnosis by identifying high-risk patients. These high-risk patients can receive CT and/or MRI inspections or more frequent follow-up monitoring and achieve early diagnosis of HCC.

In conclusion, we established a simple, efficacious ratio APAR to predict HCC from the healthy and CHB patients, especially those with low even normal AFP levels. A combination of APAR and ultrasonography can be recommended as surveillance modality for early diagnosis of HCC in clinical practice, especially in CHB patients with high APAR values whose ultrasonic imaging of lesion is indistinguishable. Our study included a training phage and a validation phase to ensure the persuasiveness of our results, however, a further study will still be conducted to optimize APAR and amend its application scope and sensitivity, and in this way, it is believed, that an optimal APAR is certain to be more helpful for the effective early HCC diagnosis and preferable prognosis.

## Patients and Methods

### Patients

The retrospective study included 463 HBV-related HCC patients, CHB patients and healthy participants recruited from Provincial Hospital Affiliated with Shandong University. The inclusion criteria for HCC patients were as follows: (1) The HCC was defined by histology, computed tomography (CT) or magnetic resonance imaging (MRI);(2) All of the HCC patients had the history of HBV infection, instead of other hepatitis virus or other factors which can cause liver damage such as alcohol, drug hepatotoxicity and autoimmune liver disease, (3) The HCC patients had no prior anti-cancer treatment, such as hepatectomy, hepatic transplantation, TACE, radiofrequency ablation, or cytokine induced killer cell therapy. The selection criteria for CHB patients included that (1) CHB was diagnosed by the presence of the HBV DNA in polymerase chain reaction assays; (2) All of the patients had no related treatment for at least half a year before the recruitment; (3) They had no other factors except for HBV which damaged the liver. Participants with insufficient medical records were excluded. To identify and validate a predicting index for HBV- related HCC from CHB patients and the healthy, the investigational cohort was randomly divided into the training set (n = 224) and the validation set (n = 239).

### Ethical approval and informed consent

The study protocol was approved by Ethics Committee of Shandong Provincial Hospital. Informed consent was obtained according to the committee’s regulations. All methods were performed in accordance with the relevant guidelines and regulations of the committee.

### Study design

463 participants meet the predetermined criteria were included in this study. The demographics, laboratory variables and clinicopathological data were collected from electronic medical records in the hospital. We firstly analyzed the routine laboratory variables of 224 patients in the training set, and then the performance of our results were validated in an independent cohort of 239 patients in the validation set.

#### Training phase

First, we collected demographics and laboratory variables of 224 participants in our study including 76 HCC patients, 53 CHB patients and 95 healthy participants. 12 laboratory variables were analyzed in the univariate analysis. 8 variables that were differently expressed between HCC and non-cancer control (healthy and CHB) then entered the stepwise multivariate analysis. 5 independent risk factors were screened out to construct the logistic regression model for differentiation of HBV-related HCC group and non-cancer control group. For simplification, the 5-variable panel was simplified to a 3-variable panel without losing accuracy after repeated verification. And then, we further identified a simplified index containing 3 variables as handy marker for diagnosis of HCC in clinical routine use.

#### Validation phase

To further validate diagnostic efficacy of the simplified index for prediction of HCC, we recruited another independent cohort of 239 participants for the validation phase analysis. The selection criteria for HCC and CHB patients were consistent with previous narrative. The age, gender and composition of disease were identical with the training set. We also got their demographics and laboratory variables from their electric medical records.

### Statistical analysis

Statistical analysis was performed by SPSS software version 22.0 (SPSS Inc., Chicago, IL, USA). Data were expressed as mean ± SD unless otherwise stated. Differences of continuous variables between groups (HCC versus non-cancer control, HCC versus healthy, HCC versus CHB) were assessed by Mann-Whitney U test, whereas categorical variables were compared by χ^2^ or Fisher exact tests. A two sided P < 0.05 was considered statistically significant. The univariate analysis was performed between the HCC and non-cancer group including the following 12 routine laboratory variables: WBC count, RBC count, PLT count, lymphocyte count, monocyte count, neutrophil count, AFP, AST, ALT, GGT, ALP, TBIL. Significant variables (P < 0.05) from the univariate analysis were accessed into multivariate analysis by a forward logistic regression to identify independent risk factors, and independent risk factors were used to construct logistic model. The predicted probability of predicting HCC was used to construct ROC curve. The diagnostic efficacy of each panel was assessed by AUC. The optimal cut-off values for diagnosis were selected using Youden’s index, which were maximal values at the sum of the sensitivity and specificity. The best panel for HCC diagnosis constructed from the training set was applied into the validation set. Likewise, AUC was used to test the diagnostic efficiency.

### Data availability statement

The datasets generated and analysed during the current study are available from the corresponding authors on reasonable request.

## Electronic supplementary material

A Simple Noninvasive Index Can Predict Hepatocellular Carcinoma in Patients with Chronic Hepatitis B
