# Supplementary material for: A Simple Noninvasive Index Can Predict Hepatocellular Carcinoma in Patients with Chronic Hepatitis B
Source: Sci Rep. 2017 Aug 21;7:8954. doi: 10.1038/s41598-017-09358-z (PMC5567190; doi:10.1038/s41598-017-09358-z)
Supplement: Supplementary file 1 — A Simple Noninvasive Index Can Predict Hepatocellular Carcinoma in Patients with Chronic Hepatitis B [file 41598_2017_9358_MOESM1_ESM.pdf]

A Simple Noninvasive Index Can Predict Hepatocellular Carcinoma in Patients with Chronic Hepatitis B

Lihui Zhu[1, 2+], Tao Li[3+], Xiaomin Ma[4], Yumin Qiu[1], Xiaoxiao Ma[4], Yueke Lin[4], Lihui Han[4], Chengyong Qin[2]*

[1]Shandong University School of Medicine, Jinan 250012, China
[2]Department of Gastroenterology, Provincial Hospital Affiliated to Shandong University, Jinan 250021, China
[3]Department of Infectious diseases, Provincial Hospital Affiliated to Shandong University, Jinan 250021, China
[4]Department of Immunology, Shandong University School of Medicine, Jinan 250012, China
*Corresponding Author:
Chengyong Qin, M.D., Ph.D.,
Department of Gastroenterology, Provincial Hospital Affiliated to Shandong University, 324 Jingwu Road, Jinan 250021, China.
Phone: 86-531-68778250. Fax: 86-531-68776456. E-Mail: qchengy@163.com
[+] Lihui Zhu and Tao Li contributed equally to this work.

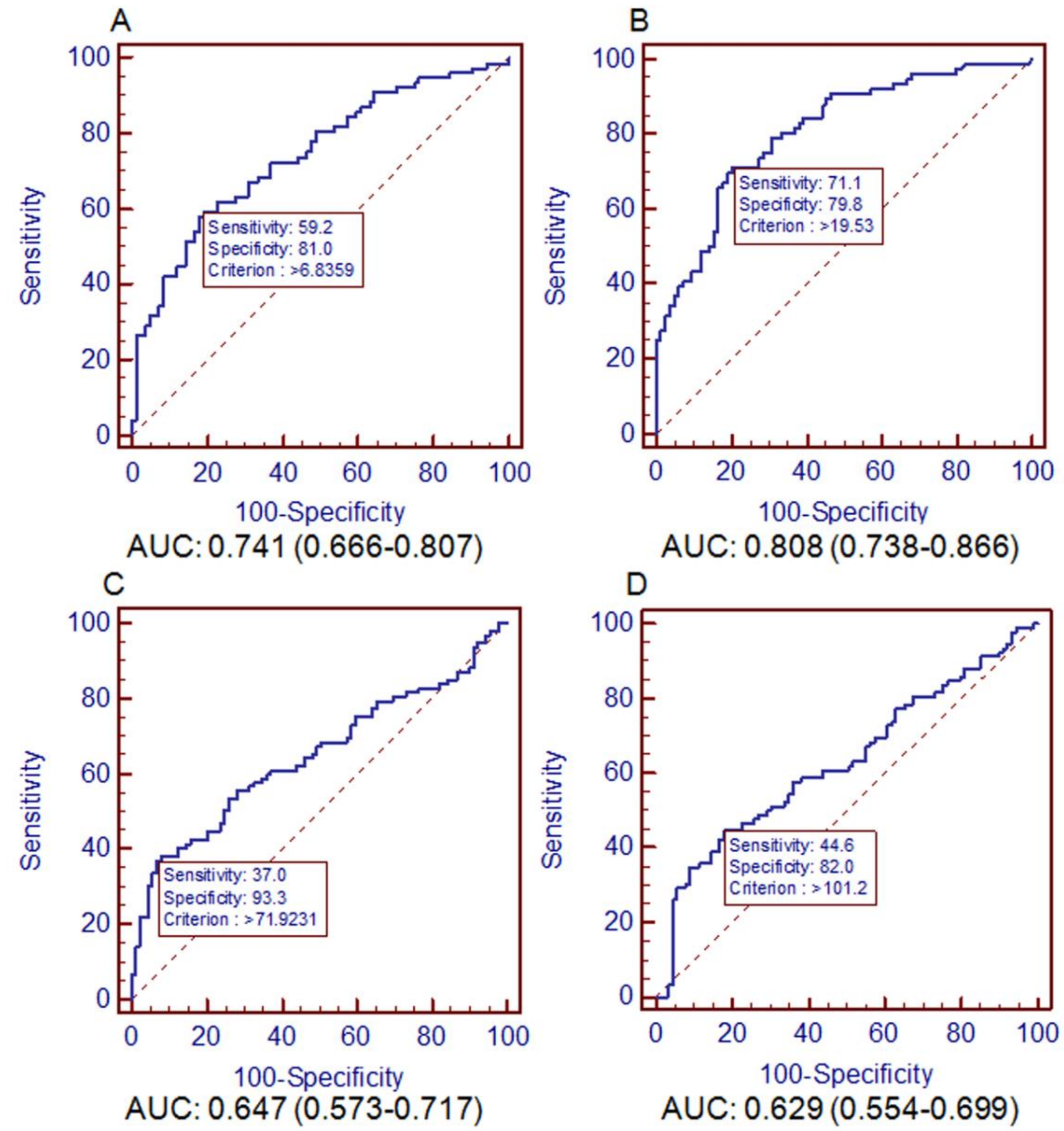

Supplement Figure S1. ROC analysis of APAR and AFP for predicting HCC from patients with cirrhosis. (A) AUC of APAR for predicting HCC from patients with cirrhosis in the training set, (B) AUC of AFP for predicting HCC from patients with cirrhosis in the training set, (C) AUC of APAR for predicting HCC from patients with cirrhosis in the validation set, (D) AUC of AFP for predicting HCC from patients with cirrhosis in the validation set.
